# Australian GP management of osteoarthritis following the release of the RACGP guideline for the non-surgical management of hip and knee osteoarthritis

**DOI:** 10.1186/s13104-015-1531-z

**Published:** 2015-10-05

**Authors:** Martin Basedow, Helena Williams, E. Michael Shanahan, William B. Runciman, Adrian Esterman

**Affiliations:** School of Psychology, Social Work and Social Policy, University of South Australia, Adelaide, SA Australia; School of Medicine, Flinders University, Adelaide, SA Australia; Southern Adelaide Local Health Network, Adelaide, SA Australia; Australian Institute of Health Innovation, Macquarie University, Sydney, NSW Australia; Centre for Population Health Research, University of South Australia, Adelaide, SA Australia; The Joanna Briggs Institute, University of Adelaide, Adelaide, SA Australia; Sansom Institute of Health Service Research and School of Nursing and Midwifery, University of South Australia, Adelaide, SA Australia; Centre for Chronic Disease Prevention, James Cook University, Cairns, QLD Australia

**Keywords:** Osteoarthritis, General practitioners, Primary health care

## Abstract

**Background:**

Osteoarthritis (OA) is a highly disabling and costly condition with an escalating prevalence in Australia due to the ageing and increasing obesity of the population. The general practitioner (GP) plays a central role in the management of this condition. The aim of this study was to examine opinions about the management of OA by Australian GPs following the release of the Royal Australian College of General Practitioners Guideline for the non-surgical management of hip and knee OA (RACGP OA CPG), and to compare the results with an earlier survey administered by the National Prescribing Service.

**Methods:**

In January 2013, a self-administered questionnaire was sent to 228 GPs to determine their treatment approaches to OA management using a clinical vignette of a patient with OA. This was compared with results from a similar survey undertaken in 2006.

**Results:**

Seventy-nine GPs returned questionnaires (response rate 35 %). GP recommendations for paracetamol, a paracetamol/codeine compound, and oral non-steroidal anti-inflammatory drugs (NSAIDs) were consistent with recommendations in the RACGP OA CPG, and varied little from the previous survey. Notably, there was a marked increase between surveys in GP recommendations for tramadol (p = 0.004) and more potent opioids (p < 0.001). Advice about the adverse effects of NSAIDs and codeine and how to manage them increased between surveys (p = 0.038 and 0.005, respectively). For all non-pharmacological treatments, there were only minor changes in the percentage of GP recommendations when compared with the previous survey, however they remain underutilised.

**Conclusions:**

GPs generally demonstrated a conservative approach to the treatment of OA, however, the increased recommendations for more potent opioids warrants further investigation. Patients should be made aware of the risks of medications through the use of decision aids, which can provide structured guidance to treatment. Non-pharmacological interventions were not given the importance that is suggested by clinical practice guidelines.

## Background

Osteoarthritis (OA) is a chronic disease affecting more than 1.9 million Australians (nearly 9 % of the population) with an annual health cost estimated at AUD$3.7 billion [1]. Prevalence surveys suggest that more than 50 % of the population aged over 65 have radiological evidence of OA, whilst it is universally present amongst those aged over 85 [2]. It is the sixth most common condition managed by General Practitioners (GPs) in Australia, accounting for 2.8 % of encounters [3], yet there is a paucity of detailed information about GP OA management [4].

Despite a multitude of clinical practice guidelines (CPGs) for OA that have been developed both locally, nationally and internationally, there is abundant evidence of suboptimal OA care [5]. Furthermore, the uptake of CPGs by health professionals is highly variable [6], most likely due to a range of barriers that have been variously ascribed to organisational, clinician and patient factors [7].

The aim of our present study was primarily to examine opinions about the management of OA by Australian GPs following the release in 2009 of the Royal Australian College of General Practitioners Guideline for the non-surgical management of hip and knee OA (RACGP OA CPG) [8]. To our knowledge, no other studies have evaluated the impact of CPGs on the management of OA. A secondary objective was to compare these results with those from an earlier survey reported by the National Prescribing Service (NPS) [9].

## Methods

This paper provides the results of a new GP survey and then compares them to the results of a previously published survey.

### Previously published NPS cross-sectional survey (2006)

The survey was conducted by the National Prescribing Service Limited.

#### Target population

All Australian GPs.

#### Recruitment

The NPS did not publish recruitment details in their report and did not respond to requests for this information.

#### Survey instrument

**Fig. 1:**
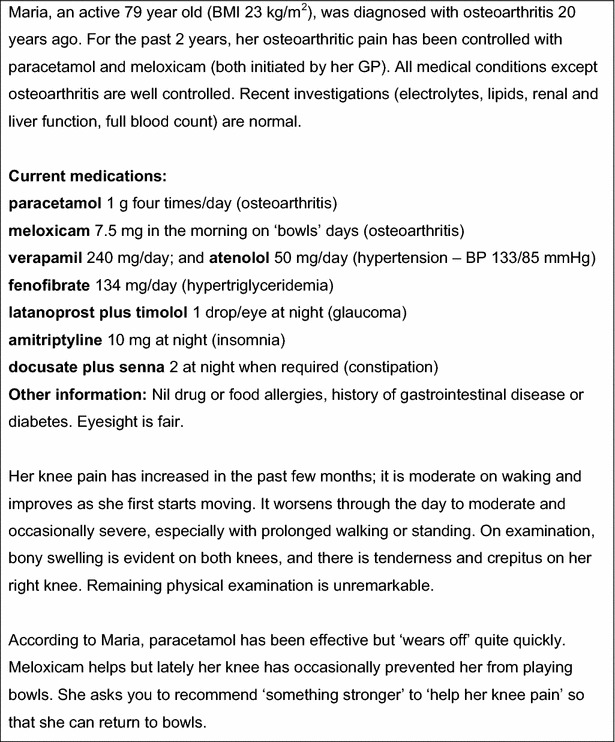
Clinical vignette

The questionnaire used a clinical vignette of a hypothetical 79 year old woman with moderate to severe OA (see Fig. 1). Respondents were asked to describe what non-pharmacological therapy they would initiate for her OA, the analgesic regimen they would use, and what information about analgesic use they would provide her with. Response options to the 4-part questionnaire included yes/no answers and 1–4 line open-ended text.

### Current CTA cross-sectional survey (2013)

#### Target population

Australian GPs from New South Wales and South Australia.

#### Recruitment

The survey was conducted of GPs who had consented to participate in the CareTrack Australia (CTA) study, a population based study of the appropriateness of care [10]. GPs had been initially identified by randomly selected CTA patients who had been asked to name their treating GP for at least one of 22 selected conditions for the period 2009–2010, one of which was OA. The subset of GPs treating CTA patients for OA was the target population. The survey was mailed to GPs in December 2012, with AUD$150 paid as an incentive for completion. Reminder letters were sent with a set completion deadline of 31 January 2013. The return of questionnaires was considered to be implied consent to take part in the study.

#### Survey instrument

The new survey used the NPS questionnaire, modified to include two additional questions relating to GP workload.

### Statistical analysis

Given a population of approximately 29,000 GPs in Australia, the response of 79 provides, at worst, ±11 % accuracy for any dichotomous questionnaire item with 95 % confidence. Descriptive data are presented as counts and percentages, with ninety-five percent confidence intervals. Comparison of results between the two surveys, and between GP demographics of the current survey and the Australian GP population was undertaken using Chi squared tests. All statistical analyses were undertaken using the Statistical Package for Social Scientists, version 21 (SPSS, Chicago, IL, USA).

### Ethics approval

Ethics approval was obtained from the Human Research Ethics Committee of the University of South Australia.

## Results

### Characteristics of respondents

There were 79 responses to a mail-out of 228 questionnaires (response rate 35 %). Respondents were marginally older than the Australian GP population, the gender composition was identical, and their practice size was larger (see Table 1). The mean number of years since graduation was 28.2 (SD = 10.5), the mean number of OA patients seen per week was 21.1 (SD = 16. 9) and the mean weekly hours worked in clinical practice was 34.2 (SD = 13.7).

**Table 1:** Gender, age group and practice size of GP respondents

	Respondents		Australian GP population [3, 34, 35]		Signif.
	n	%	n	%	
Gender					0.921
Female GPs	33	42	12,095	42	
Male GPs	46	58	16,916	58	
Total	79	100	29,011	100	
Age group					0.009
<44	15	19	9831	33	
45–64	48	61	15,449	54	
65 or greater	16	20	3731	12	
Total	79	100	29,011	100	
Practice size					<0.001
Solo	2	3	2456	35	
2–5	22	29	3075	44	
6 or greater	52	68	1504	21	
Total	76	100	7035	100	

### Pharmacological therapies

Table 2 shows the pharmacological therapies recommended for the hypothetical patient vignette.

**Table 2:** Recommended pharmacological therapies for the hypothetical patient vignette

Drug	Number and percentage of respondents^a^ (n = 200)			Number and percentage of respondents^a^ (n = 79)			Signif.
	2006			2013			
	n	%	95 % CI	n	%	95 % CI	
Paracetamol (including slow release option)	190	95	91–97	72	91	83–96	0.349
Oral NSAID (conventional or COX-2 selective)	160	80	74–85	66	84	74–91	0.610
Topical NSAID (conventional or COX-2 selective)	19	10	6–14	1	1	0–6	0.032
Tramadol	29	15	10–20	24	30	21–42	0.004
Opioids–paracetamol plus codeine	28	14	9–20	14	18	10–28	0.550
Opioids–codeine	65	33	26–39	5	6	2–14	<0.001
Opioids–oxycodone, morphine or buprenorphine	10	5	3–9	16	20	12–31	<0.001
Intra-articular injections	12	6	4–10	8	10	5–19	0.344
Glucosamine and/or chondroitin	78	39	33–46	10	13	6–22	<0.001
Fish or krill oil	11	6	3–9	10	13	7–22	0.074

^a^Respondents may have more than one response

Given that the vignette stated that the patient’s usual paracetamol and meloxicam (7.5 mg a day) drug therapy was ineffective, three quarters of respondents (75 %) recommended a change to slow release paracetamol tablets (2 × 665 mg) to be taken 3 times a day. Nearly all GPs (96 %) selected paracetamol or the compound paracetamol/codeine medication. All GPs who chose codeine (mainly 30 mg, as needed) also selected paracetamol.

In our 2013 survey, a high percentage of GPs (86 %) suggested supplementing paracetamol (often with codeine) with NSAIDs. In recommending NSAIDS, over two-thirds of GPs (67 %) chose meloxicam (mainly 7.5 mg, once daily when required). Ten percent selected other NSAIDs (including ibuprofen, diclofenac and naproxen) and 6 % selected other COX-2 selective NSAIDs such as celecoxib. Only one GP nominated a topical NSAID.

Notably, tramadol was recommended by nearly one-third of GPs (30 %) with 20 % choosing 50–100 mg (2–4 times daily when required) and 10 % selecting the slow release option (100–150 mg once or twice daily, regularly). In all responses, tramadol was to be used with paracetamol.

Nine percent of GPs selected oxycodone and 11 % selected buprenorphine or fentanyl patches, with 3 % (n = 2) choosing to solely use these opioids. All GPs who recommended these stronger opioids acknowledged the risks associated with their use (potentially addictive, and may cause constipation and confusion).

Thirteen percent of GPs chose glucosamine and/or chondroitin. In all instances, these complementary and alternative medicines (CAM) were selected together with paracetamol and an NSAID. Of the miscellaneous pharmacological options selected by GPs, fish or krill oil was chosen by 13 % of GPs, and intra-articular corticosteroid injection by 10 %. No GPs nominated viscosupplements (hyaluronic acid).

The major differences between the 2006 NPS survey and the 2013 CTA survey were increases in the recommended use of tramadol (p = 0.004) and more potent opioids (oxycodone, morphine and buprenorphine) (p < 0.001), and a decrease in recommendations for glucosamine and chondroitin (p < 0.001), topical NSAIDS (p = 0.032) and codeine (p < 0.001).

### Non-pharmacological therapies

Table 3 shows the non-pharmacological therapies recommended for the hypothetical patient vignette.

**Table 3:** Recommended non-pharmacological therapies for the hypothetical patient vignette

Therapy	Number and percentage of respondents^a,b^ (n = 193)			Number and percentage of respondents^a,b^ (n = 78)			Signif.
	2006			2013			
	n	%	95 % CI	n	%	95 % CI	
Physiotherapy	115	60	53–66	42	54	42–65	0.451
Land-based exercise	77	40	33–47	36	46	35–58	0.610
Hydrotherapy	78	40	34–47	24	31	21–42	0.179
Heat and cold therapies	57	30	24–36	16	21	12–31	0.173
Referral for joint replacement	37	19	14–25	13	17	10–26	0.758
Knee taping/bracing and insoles	21	7	4–12	5	6	2–14	0.366
Weight loss	15	8	5–13	4	5	1–13	0.611
Tai chi	^c^	^c^		4	5	2–12	^c^
Acupuncture	13	7	4–11	3	4	1–11	0.529

^a^Respondents may have more than one response

^b^There was an occasional missing value

^c^Unknown

Over half the respondents (56 %) recommended one or two non-pharmacological therapies and 44 % selected more than two. The most commonly recommended combination was physiotherapy and exercise therapy including hydrotherapy (33 %). Heat therapy for symptomatic OA relief was universally favoured over cold therapy (21 vs. 0 %). Several GPs (17 %) nominated referral for joint replacement.

There were only minor changes in recommendations for non-pharmacological therapies between the two surveys, and none were statistically significant.

### GP advice to patients

Table 4 presents a summary of recommended advice to be provided to the patient about analgesic use.

**Table 4:** Recommended information to be provided to patient about analgesic use

Information	Number and percentage of respondents^a,b^ (n = 199)			Number and percentage of respondents^a,b^ (n = 70)			Signif.
	2006			2013			
	n	%	95 % CI	n	%	95 % CI	
Dosing advice							
Taking medication regularly by clock	126	63	56–70	27	39	28–50	<0.001
Use analgesics pre-emptively (e.g. before playing bowls or before strenuous exercise)	33	17	12–22	6	9	4–17	0.150
NSAID to be taken on an ‘as required’ basis	24	12	8–17	5	7	3–16	0.359
When and how to take analgesics on an ‘as required’ basis	13	7	4–11	3	4	1–12	0.697
NSAID to be taken with or after food	12	6	3–10	3	4	1–12	0.807
Maximum of 4 g of paracetamol in a day	7	4	2–7	1	1	0–8	0.634
Ensure adequate dosage of analgesics is taken	7	4	2–7	6	9	4–17	0.170
Paracetamol Slow Release offers a more convenient option if preferred	7	4	2–7	0	0	0	0.249
Use lowest effective dose	6	3	1–6	6	9	4–17	0.110
Awareness and management of adverse effects							
Adverse effects of medications (in general)	35	18	13–23	5	7	3–16	0.055
Adverse effects of NSAIDs (e.g. gastrointestinal bleeding, increased blood pressure)	32	16	12–22	20	29	19–40	0.038
Potential opioid-induced constipation and strategy for managing constipation	30	15	11–21	6	9	4–17	0.242
Adverse effects of codeine (e.g. constipation, risk of dependency)	9	5	2–8	11	16	9–26	0.005
Risk of drowsiness with opioid medications	6	3	1–6	3	4	1–12	0.610
Follow-up							
Regular follow-up to assess efficacy and titrate doses	23	12	8–17	5	7	3–16	0.416
Potential drug interactions							
Avoid other preparations containing paracetamol	10	5	3–9	1	1	0–8	0.339
Drug interactions (in general)	6	3	1–6	1	1	0–8	0.779
Check with health professional before taking over-the-counter medications	5	3	1–6	2	3	1–10	0.779
Role and place of different analgesics							
Options of stronger analgesics if required in future	18	9	6–14	22	31	22–43	<0.001
Paracetamol is the safest option	13	7	4–11	18	26	17–37	<0.001

^a^Respondents may have more than one response

^b^There was an occasional missing value

In the 2013 survey, GPs were much less likely to include the need to take medications regularly (p < 0.001). Advice about the adverse effects of NSAIDs and codeine and how to manage them increased from 21 % in 2006 to 45 % in 2013 (p < 0.001). GPs were far more likely to consider recommending stronger analgesics if needed in future (p < 0.001). Nonetheless, paracetamol was still considered the safest option (p < 0.001). In both surveys, very few GPs chose to advise the patient about the need to avoid other preparations containing paracetamol and the importance of checking with health professionals before taking over-the-counter medications as a safeguard against potential drug interactions.

## Discussion

### Pharmacological therapies

The scenario provided was for an elderly lady who was currently on paracetamol and meloxicam for her OA. She also suffered from insomnia, constipation, hypertension, hypertriglyceridemia and glaucoma. Interestingly, relatively few GP responses discussed these comorbid conditions, or commented on how these might impact on her treatment, despite the importance placed on such factors by recent guidelines [11].

Paracetamol and a paracetamol/codeine combination used in an optimised and regular dosage regimen were the primary analgesics of choice recommended by GPs (96 %), although evidence suggests that patients may underestimate the value of paracetamol due to a perception of ineffectiveness and a failure to appreciate the benefits of long-term use [12]. In view of this, GPs have a crucial role in educating patients about the pivotal role of paracetamol in ameliorating pain.

NSAIDs were recommended by most GPs (86 %), who also recognised the importance of monitoring the risk factors associated with their use. Combining an NSAID with paracetamol—as most GPs recommended, allows the use of a lower NSAID dose, thereby reducing the risk of adverse effects [13]. Whilst there is no evidence to support one oral NSAID over another with regard to efficacy [14], it was notable that fewer GPs endorsed the use of topical NSAIDs when compared with the previous survey, despite evidence supporting their effectiveness, particularly for those aged over 75 [15]. The underutilisation of topical NSAIDs has been previously reported [16] and is consistent with the findings of the CTA study [10].

Recommendations by GPs for tramadol use have doubled since the 2006 NPS survey [17], reflective of its effectiveness in treating moderate to severe pain. Nevertheless, adverse drug reactions are common, particularly in the elderly, and the potential for serious drug–drug interactions should not be underestimated [18].

The GPs in our survey recommended using more potent opioids (oxycodone, morphine and buprenorphine), and this has also increased significantly since the previous survey [17]. In addition to modest benefits and the high risk of adverse effects, their use was not indicated based on the clinical scenario provided. According to the RACGP OA CPG, more potent opioids should be reserved for severe OA when joint-replacement surgery is delayed or contraindicated [8]. Researchers have elsewhere commented on the increasing level of GP prescribing of opioids in OA management [19]; further investigation is clearly warranted.

Glucosamine and chondroitin are the most commonly used CAM treatments for OA [20], however, there is no evidence of their clinical efficacy. Recent RCTs have shown that glucosamine has a similar effect to placebo on pain, with commercially funded trials having larger effects than industry-independent trials [21]. The percentage of GPs recommending their use was only one-third of that in the previous NPS survey [17], perhaps reflecting an increased awareness by GPs of their ineffectiveness.

A high percentage of GPs recommended fish oil and krill oil in spite of the fact that their early promise has not been realised, and there is no evidence of clinical efficacy in well-designed studies [22]. No herbal therapies were recommended by GPs.

Intra-articular corticosteroid injections can provide rapid symptomatic relief, however, their short benefit duration (up to 4 weeks) and cost may limit their value for chronic diseases such as OA [23]. The recent deletion of the scheduled item number (50124) for joint injection by Medicare, the Australian government’s medical insurance scheme for primary health care, may have influenced the GPs’ decisions not to recommend this therapy. Additionally, lack of training or confidence in their ability to perform intra-articular injections, or a belief in the superiority of patient outcomes with image guided injections, may have affected their choice.

### Non-pharmacological therapies

For all non-pharmacological treatments, there were only minor changes in the percentage of GP recommendations when compared with the 2006 survey. A majority of GPs recommended a multi-disciplinary approach incorporating various combinations of physiotherapy, exercise and hydrotherapy, although these interventions were not given the importance that is suggested by the RACGP OA CPG. For example, although exercise has similar effect sizes to simple analgesic and NSAIDs but with fewer contraindications or adverse effects [21], it was recommended by only just over half the GPs. Previous research has highlighted the gap between evidence-based recommendations for care and the uptake of non-pharmacological interventions [24]. In a recent large scale cross-sectional study of GP management of hip and knee OA in Australia, Brand and colleagues confirmed the suboptimal use of non-pharmacological therapies for OA [4].

While there is some evidence that the Australian government’s introduction of GP funding incentives for chronic disease management involving allied health professionals has been associated with positive outcomes [25], there clearly is scope for greater use of non-pharmacological therapies, in line with CPG recommendations.

Heat therapy was supported by around 1 in 5 GPs notwithstanding the lack of evidence for its efficacy. Its role in reducing pain and enabling a resumption of physical activity may be seen by GPs as being helpful. There was no support amongst surveyed GPs for cold therapy, despite some evidence that it reduces swelling, and improves range of motion, knee strength and function [26].

The RACGP OA CPG only cautiously recommended tai chi [8], however, Arthritis Research UK have recently reported a number of randomised controlled trials that support its effectiveness in treating OA of the knee [27]. Only 5 % of GPs in our survey recommended that their patients initiate tai chi.

Several GPs considered acupuncture as a worthwhile option, but it has been reported that its putative benefits are most likely attributable to placebo effects [28]. A recent CPG issued by the American College of Rheumatology, however, conditionally recommended acupuncture for patients with moderate to severe pain and for whom surgery is either not desired or is contraindicated [15].

### GP advice to patients

The provision of inadequate analgesic information to OA patients by GPs has been previously reported [29]. In the current study, GPs emphasised the importance of advice on appropriate dosing, yet research has highlighted the reluctance by patients to take painkillers at the prescribed dosage and frequency [30]. Whilst there has been a noticeable improvement between the 2006 and 2013 surveys in the percentage of GPs providing advice about the adverse effects of NSAIDs and codeine, there is evidence that many patients still don’t adequately understand the risks associated with use [31]. Shared decision making between patients and their GPs through the use of decision aids should be encouraged. The Cochrane Musculoskeletal Group, for example, has developed easy-to-use decision algorithms which prepare patients for consultations by explaining options, quantifying the risks and benefits and providing structured guidance to treatment [32]. The challenge for GPs is how best to integrate such tools into routine care, which will necessitate changing some work practices, and will inevitably be inconvenient for busy clinicians [33].

### Limitations

There were several limitations to our study. Firstly, because of the comparatively small sample size, the generalizability of results should be treated with caution. GPs in our study were found to be broadly similar in terms of gender, however, they were relatively older than the Australian GP population and general practices in our study were larger.

Secondly, the 35 % response rate to our survey was low. Although poor response rates have been observed in many GP studies [36], there was clearly a potential for selection bias.

Finally, there were some difficulties with our study associated with use of the 2006 NPS survey. For example, we were unable to obtain the recruitment methods for this survey, nor did we have any demographic details of survey participants. This limited our ability to make further comparisons between the 2006 and 2013 surveys. There were also no questions in the NPS survey that related to the provision of self-management and lifestyle advice, which are recognized in CPGs as essential features of appropriate OA management.

## Conclusions

Despite the existence of CPGs for the best practice management of OA, our study has highlighted a diversity of therapeutic approaches for a typical case. There have been some change in GP treatment recommendations following the release of the RACGP OA CPG, however, it is not possible to infer a causal relationship. Tramadol and more potent opioids appear to be more commonly favoured than previously; the latter, in particular, warrants further investigation, as its use does not appear to be in accord with CPG recommendations. Furthermore, whilst there have been only minor changes in recommendations for the non-pharmacological treatment of OA, it would appear that GPs are still not giving these options the attention that is warranted.

## Abbreviations

OA: osteoarthritis
GP: general practitioner
RACGP: Royal Australian College of General Practitioners
NPS: National Prescribing Service
NSAID: non-steroidal anti-inflammatory drug
CPGs: clinical practice guidelines
CTA: CareTrack Australia
CAM: complementary and alternative medicine
RCT: randomised controlled trial
